# A Comprehensive Investigation into the Distribution of Circulating B Cell Subsets in the Third Trimester of Pregnancy

**DOI:** 10.3390/jcm11113006

**Published:** 2022-05-26

**Authors:** Ágnes Kövér, Rudolf Lampé, Krisztina Szabó, Tünde Tarr, Gábor Papp

**Affiliations:** 1 Department of Obstetrics and Gynecology, Faculty of Medicine, University of Debrecen, H-4032 Debrecen, Hungary; agi.kover@gmail.com (Á.K.); rudolflampe@msn.com (R.L.); 2 Division of Clinical Immunology, Department of Internal Medicine, Faculty of Medicine, University of Debrecen, H-4032 Debrecen, Hungary; krisztinaszabo@med.unideb.hu (K.S.); tarr.tunde@med.unideb.hu (T.T.)

**Keywords:** pregnancy, B cell subsets, regulatory B cells, interleukin-10

## Abstract

Maternal B cells play a crucial role in the development and maintenance of pregnancy, due to their humoral activities and regulatory functions. In the study, we investigated the alterations in the distributions of naïve and memory B cell subsets, as well as regulatory B (Breg) cells, in the third trimester of pregnancy. Peripheral blood from 14 healthy pregnant women in the third trimester and 7 healthy non-pregnant women was collected and examined for the frequencies of B cell subsets, including IgD^+^CD27^−^ naïve, IgD^+^CD27^+^ un-switched memory, IgD^−^CD27^+^ switched memory, CD38^int^CD24^int^ mature–naïve, CD38^−^CD24^hi^ primarily memory and CD38^hi^CD24^hi^ transitional B cells by flow cytometry. Breg cell subsets were also characterized based on the expression of CD5, CD1d and IL-10. In pregnant women, the proportions of un-switched memory and transitional B cells were significantly decreased. Additionally, the frequencies of both CD5^+^CD1d^+^ Breg and IL-10-producing B10 cells were decreased in pregnancy. Changes in the distribution of transitional B cells as well as Breg cells may be crucial contributors for the development of altered maternal immune responses and tolerance needed for the maintenance of normal pregnancy in the third trimester.

## 1. Introduction

Various abnormalities of immune responses have been reported in the pathophysiological backgrounds of the most common pregnancy complications, including implantation failure, recurrent pregnancy loss, preterm birth and preeclampsia [1,2,3,4,5]. These observations shed light on the importance of the immunological factors of human reproduction. Importantly, pregnancy represents a very special immunological state for women, since the maternal immune system has to tolerate the presence of the semi-allogeneic fetus to avoid its rejection. Therefore, fundamental changes are needed for developing a new order of immune mechanisms, which should be unresponsive to the paternal antigens of the haploidentical fetus that are recognized as foreign; while, at the same time, they have to protect the human body against pathogens. These immunological changes encompass the reinforcement of regulatory and tolerogenic activity of both the innate and adaptive immune system, and these modulations can either be local or systemic. However, the directions for these changes appear to be fundamentally different during pregnancy. Increased activation of systemic innate immunity [6] during gestation is accompanied by the phase-dependent modulation of local innate immunity, which is functionally active early in pregnancy to assist in implantation, down-regulated through most of the gestation, and then increased again with parturition [7]. During pregnancy, hormonal changes may play an important role in the recruitment of innate immune cells at the maternal–fetal interface. Accordingly, natural killer (NK) cells with the distinctive CD56^bright^CD16^−^ phenotype, as well as macrophages and dendritic cells (DCs), accumulate in uterine mucosa in early pregnancy, and contribute to decidual remodeling [8,9,10]. Regarding the adaptive immune system, an altered hormonal profile during pregnancy results in changes in T cell functions as well [11]. Previous studies have suggested that pregnancy is accompanied by a decreased Th1 and an enhanced Th2 immune response [12]. On the other hand, there is an increase in interleukin (IL)-10 and transforming growth factor (TGF)-beta expression due to the hormonal changes as well as alloantigen exposure, resulting in the expansion of regulatory T (Treg) cells in pregnancy [13,14]. A decrease in Treg cell number or function contributes to the etiology of recurrent spontaneous abortion and preeclampsia [15]. Other maternal–fetal tolerance mechanisms include an increase in the expression of the inhibitory molecule programmed death-ligand 1 (PD-L1) in trophoblastic tissue, and a lack of class I and II major histocompatibility complex (MHC) expression required for T cell activation by syncytiotrophoblast cells [16]. Although our knowledge is constantly growing, several points of this T cell regulation remain unclear, and some of the results are conflicting.

B cells also perform crucial roles during pregnancy, and a wide spectrum of changes was reported in terms of their activation, proliferation, differentiation, antibody and cytokine production, and regulating properties. These alterations are aimed at the establishment of a tolerant environment by producing protective antibodies to encounter foreign paternal antigens and by developing anti-inflammatory characteristics to sustain normal pregnancy [17]. Underlying the importance of the synchronized changes in endocrine and immune systems, pregnancy hormones contribute to the regulation of B cell populations and antibody production. Fetal trophoblasts positively regulate the generation of IL-10-producing B cells with regulative properties, related to gonadotropic hormones [10]; additionally, in pregnancy, the B cell response to mitogens and infectious agents decreases [18]. Although antibody production associated with enhanced immune activation may be harmful in pregnancy, maternal B cells can also produce protective antibodies against paternal antigens, such as asymmetric antibodies that bind paternal antigens, but do not produce responses against them [13]. Moreover, the local increase in B regulatory (Breg) cell proportions during pregnancy potentially represents another important maternal–fetal tolerance mechanism [19]. 

As for peripheral blood, a recent study reported an increase in naïve B cell proportions and a reduction in transitional and Breg cell frequencies [20]. These findings suggest the importance of the contribution of certain B cell subtypes to the development of appropriate peripheral immune tolerance in pregnancy. Therefore, our study aimed to confirm the changes described in recent studies and further investigate a wide spectrum of B cell subsets, including B cell types with regulative capacity in healthy pregnant women.

## 2. Materials and Methods

### 2.1. Study Population

Fourteen healthy pregnant women (mean age: 29.86 ± 6.18 years) in their third trimester (between week 34 and 37), who attended the Outpatient Clinic of the Department of Obstetrics and Gynecology, Clinical Centre, University of Debrecen for a routine pregnancy care examination and blood collection, were enrolled in the study. All of the pregnant women delivered in term later on (in week 37–41). The control group consisted of seven healthy non-pregnant women of reproductive age (mean age: 30.29 ± 5.79 years). No pregnant women and non-pregnant women included in this study had ongoing infections, either viral or bacterial, excluded by the absence of physical signs of infection and a white blood cell count, CRP, and urinary result within the normal range. Other exclusion criteria were a history of diabetes, diagnosed immunological diseases, preeclampsia or HELLP syndrome, use of any medication, smoking, and ongoing severe complications during the pregnancy.

### 2.2. Flow Cytometric Analysis of Different B Cell Subsets

Peripheral blood samples were collected in lithium heparin-coated BD Vacutainer tubes, and peripheral blood mononuclear cells (PBMCs) were isolated by Ficoll-Histopaque (Sigma-Aldrich, St. Louis, MO, USA) density gradient centrifugation. Cells were harvested and washed twice, then stained for 20 min at 4 °C using fluorochrome-conjugated monoclonal antibodies against cell surface markers. The following monoclonal antibodies were used: anti-IgD-fluorescein isothiocyanate (FITC) (clone: IADB6), anti-CD27-phycoerythrin (PE) (clone: 1A4CD27), anti-CD19-phycoerythrin-Cyanine dye 5 (PE-Cy5) (clone: J3-119) (Beckman Coulter Inc., Fullerton, CA, USA), anti-CD38-FITC (clone: HIT2) (BD Biosciences, San Diego, CA, USA), anti-CD24-allophycocyanin (APC) (clone: ML5) (BioLegend, San Diego, CA, USA), anti-CD1d-PE (clone: CD1d42), and anti-CD5-FITC (clone: UCHT2) (both from BD Biosciences). At least 20,000 CD19^+^ events of each sample were analyzed within the lymphocyte population. According to the cell surface markers, the following B cell subsets were identified within the CD19^+^ lymphocytes: IgD^+^CD27^−^ naïve, IgD^+^CD27^+^ un-switched memory, IgD^−^CD27^+^ switched memory, CD38^int^CD24^int^ mature–naïve, CD38^−^CD24^hi^ primarily memory, and CD38^hi^CD24^hi^ transitional B cells, in addition to CD5^+^ B-1a and CD5^+^CD1d^+^ Breg cells. Fluorescence minus one controls (FMO) were used in all procedures to determine gate settings. Stained cells were measured with a FACSCalibur^TM^ flow cytometer (Becton Dickinson, Franklin Lakes, NJ, USA), and data were analyzed using Kaluza software version 1.2a (Beckman Coulter).

### 2.3. Intracellular Cytokine Analysis by Flow Cytometry

The determination of IL-10-producing regulatory B cells (B10) was also performed by multicolor flow cytometry with an intracytoplasmic staining method. Isolated PBMCs were cultured in modified RPMI 1640 medium with GLUTAMAX^TM^-I (Life Technologies Corporation, Carlsbad, CA, USA) supplemented with 100 U/mL penicillin, 100 ng/mL streptomycin, and 10% heat-inactivated fetal calf serum (Life Technologies) at a concentration of 2 × 10^6^ cells/mL in 24-well tissue culture plates at 37 °C in 5% CO_2_ milieu. Detection of IL-10 secretion by B10 effector cells was implemented after stimulation with phorbol-12-myristate 13-acetate (PMA) (25 ng/mL) and ionomycin (1 μg/mL) (Sigma Aldrich) for 5 h. Golgi Stop brefeldin-A (10 μg/mL) (Sigma Aldrich) was included in the culture to enhance cytoplasmic IL-10 staining by blocking its protein transport through the Golgi apparatus. The IL-10 secretion of B10 and progenitor B10 (B10_PRO_) cells was carried out by adding CpG (Toll-like receptor 9 ligand, ODN 2006 type B; 0.5 μM/mL; Hycult Biotech Inc., Uden, The Netherlands) oligonucleotides to the cultures for 48 h, and the complementation of PMA/ionomycin/brefeldin-A (PIB) during the last 5 h induced both cell subsets to produce IL-10. After harvesting the cells, cell surface staining was performed with the combination of anti-CD38-FITC, anti-CD19-PECy5, and anti-CD24-APC monoclonal antibodies for 20 min at 4 °C. The cells were then fixed and permeabilized with the Intraprep^TM^ permeabilization reagent (Beckman Coulter Inc., Miami, FL, USA) according to the manufacturer’s instructions, and intracellular IL-10 cytokines were stained with an anti-IL-10-PE (clone: JES3-9D7) (BD Biosciences) monoclonal antibody. At least 5000 events in the CD19^+^ lymphocyte population were collected during the acquisition. Measurements were performed and data were collected with the FACSCalibur^TM^ flow cytometer and data were analyzed using Kaluza 1.2a software.

**Figure 1:**
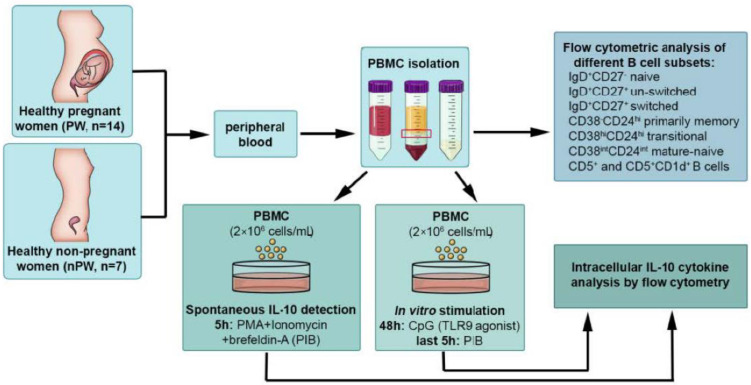
Flowchart demonstrating the study design and detailing the procedure of analyzing different B cell subsets and IL-10-producing cells by flow cytometry in healthy pregnant and non-pregnant women. PBMC, peripheral blood mononuclear cell.

The procedure of analyzing different B cell subsets and IL-10-producing cells by flow cytometry is detailed in Figure 1.

### 2.4. Statistical Analysis

Data were represented and statistically analyzed with GraphPad Prism 8 software (Graphpad Software, San Diego, CA, USA). Data are presented as mean ± SD. To assess the distribution of the data, the Shapiro–Wilk and Kolmogorov–Smirnov normality tests were used. In cases of a normal distribution, unpaired *t*-tests were used; otherwise, the Mann–Whitney U test was used. Matched data were analyzed using paired *t*-test. Differences were considered statistically significant at *p* < 0.05.

The number of enrolled individuals was estimated with a priori power calculation using G*Power v3.1.9.2. software (University of Düsseldorf, Düsseldorf, Germany) [21]. Data from previous studies with approximate changes in the percentages of transitional B and CD5 B cell subpopulations in pregnant women in their third trimester served as a basis for calculation [22,23]. In these studies, a considerably large effect size (Cohen’s d = 1.16 [22] and d = 2.91 [23], respectively) was detected in the case of the aforementioned B cell subsets between the non-pregnant and pregnant groups; thus, we enrolled individuals between 4 and 17 in each group to obtain 90% power (1 − β) and a 5% significance level (α = 0.05, two-tailed) in an unpaired *t*-test.

## 3. Results

### 3.1. The Distribution of Peripheral B Cell Subpopulations

**Figure 2:**
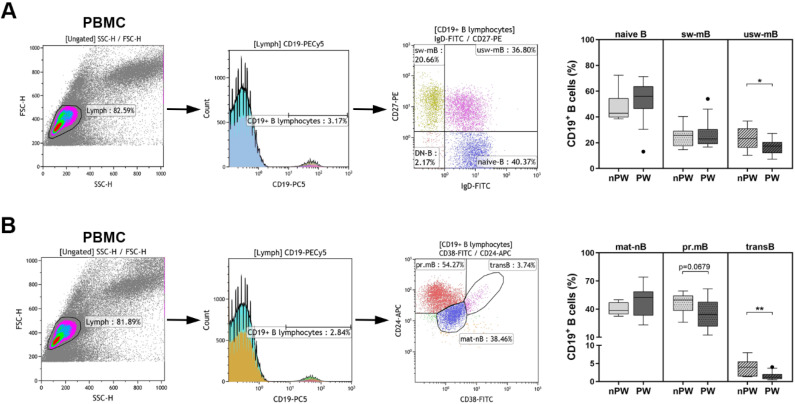
PBMCs were isolated from 14 healthy pregnant women (PW) and 7 healthy non-pregnant women (nPW), then they were stained with fluorochrome-labelled antibodies as described previously. Representative dot plots and histograms show the gating strategy of different B cell subsets (**A**,**B**). (**A**) Percentages of naïve B, switched memory B (sw-mB) and un-switched memory B (usw-mB) cells. (**B**) Frequencies of mature–naïve B (mat-nB), primarily memory B (pr.mB) and transitional B (transB) cells. Box plots represent the interquartile range (IQR) with a line in the middle as the median value. Statistically significant differences are indicated by * *p* < 0.05 and ** *p* < 0.01.

At first, the main B cell subpopulations were assessed in the peripheral blood of the enrolled pregnant and non-pregnant women. The percentages of naïve and mature–naïve B cells tended to increase in pregnant women compared to non-pregnant women, but this increase was not statistically significant (Figure 2A,B). The frequencies of un-switched memory B cells significantly decreased in pregnant women compared to those measured in the controls (23.45 ± 8.95 vs. 16.44 ± 5.75; *p* = 0.0413) (Figure 2A); moreover, the percentages of primarily memory B cells also diminished at a quasi-significant rate (46.43 ± 11.21 vs. 34.79 ± 13.93; *p* = 0.0679) (Figure 2B). The percentages of switched memory B cells did not differ in pregnant women at all in comparison with the values measured in non-pregnant women. On the other hand, the percentages of transitional B cells significantly decreased in pregnant women compared to those measured in the controls (4.15 ± 2.34 vs. 1.58 ± 1.15; *p* = 0.0021) (Figure 2B).

**Figure 3:**
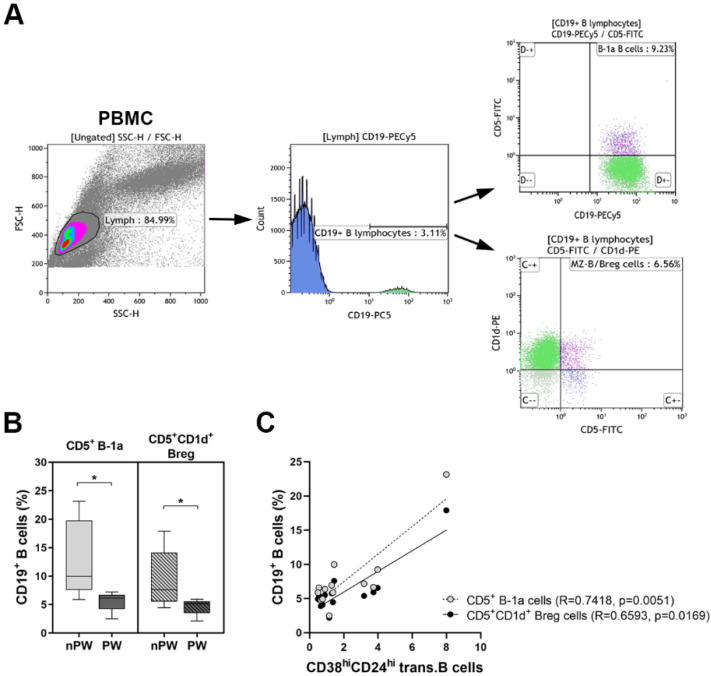
PBMCs were isolated from 14 healthy pregnant women (PW) and 7 healthy non-pregnant women (nPW), then they were stained with fluorochrome-labelled antibodies as described previously. (**A**) Representative dot plots and the histogram show the gating strategy of different CD5^+^ B-1a and CD5^+^CD1d^+^ Breg cells. (**B**) Percentages of CD5^+^ B-1a and CD5^+^CD1d^+^ Breg cells. (**C**) Correlation of the percentages of transitional B cells with CD5^+^ B-1a and CD5^+^CD1d^+^ Breg cells. Box plots represent the interquartile range (IQR) with a line in the middle as the median value. Dots indicate individual subjects. Statistically significant differences are indicated by * *p* < 0.05.

As there is no uniform consensus in Breg identification methods and the overlap between different B cell subset specific markers makes their determination even more difficult, we also measured the expression of CD5 and CD1d markers on the surface of B cells (Figure 3A). The percentages of CD5^+^ B-1a cells (12.93 ± 6.87 vs. 5.43 ± 1.74; *p* = 0.0127) and the frequencies of CD5^+^CD1d^+^ Breg cells (9.38 ± 5.22 vs. 4.53 ± 1.42; *p* = 0.0193) significantly decreased in pregnant women compared to non-pregnant controls (Figure 3B). Since human Bregs were described as being CD19^+^CD24^hi^CD38^hi^ as well, a correlation analysis was performed between the percentages of transitional B cells and B-1a or Breg cells. Both B-1a (*R* = 0.7418; *p* = 0.0051) and Bregs (*R* = 0.6593; *p* = 0.0169) showed a significant positive correlation with CD24^hi^CD38^hi^ B cells (Figure 3C).

### 3.2. The Determination of IL-10-Producing B Cells

**Figure 4:**
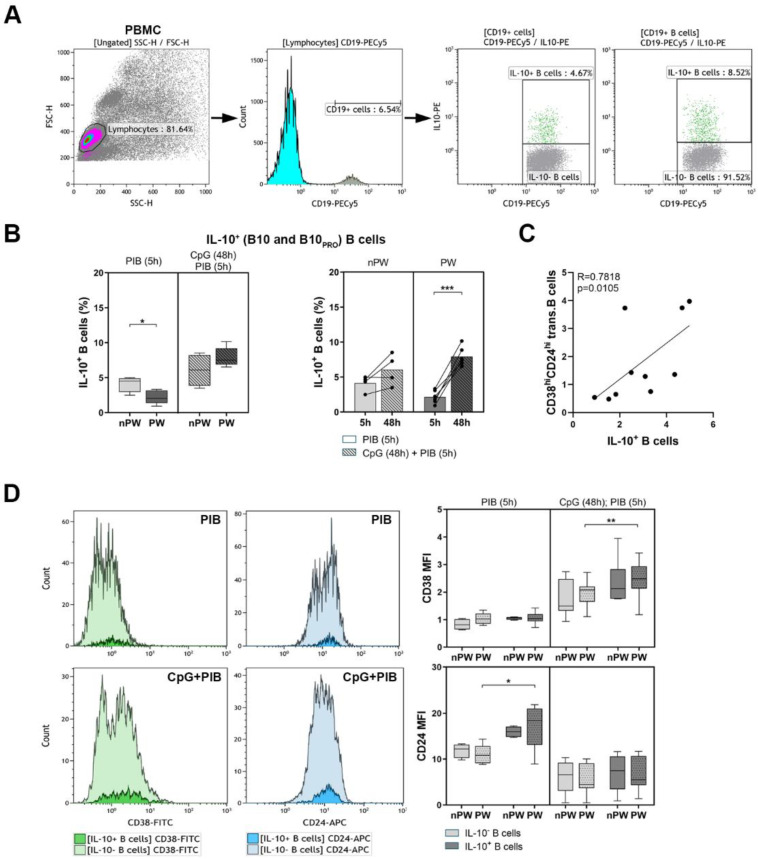
PBMCs were isolated from 6 healthy pregnant women (PW) and 4 healthy non-pregnant women (nPW), then they were stimulated with PMA/ionomycin/brefeldin-A (PIB) for 5 h and CpG-ODN for 48 h followed by restimulation with PIB for the last 5 h. (**A**) Representative dot plots and the histogram show the gating strategy of IL-10-producing B cells. (**B**) The percentages of IL-10^+^ B cells (B10 and B10_PRO_) after the stimulation with PIB and CpG-ODN + PIB. (**C**) Correlation analysis between the percentages of transitional B cells and IL-10-producing B cells. (**D**) The expression of CD38 and CD24 median fluorescence intensity (MFI) markers within IL-10^+^ and IL-10^−^ B cell subpopulations after stimulation with PIB and CpG-ODN + PIB. Box plots represent the interquartile range (IQR) with a line in the middle as the median value. Dots indicate individual subjects. Statistically significant differences are indicated by * *p* < 0.05; ** *p* < 0.01; *** *p* < 0.001.

Human PBMCs were stimulated with PMA and ionomycin for 5 h, and a known IL-10-inducing TLR9 stimulus CpG-ODN was applied for 48 h to analyze the distribution of B10 cells (Figure 4A). In the case of the PIB stimulated condition, significantly decreased percentages of B10 cells were detected in pregnant women compared to non-pregnant women (4.12 ± 1.12 vs. 2.15 ± 0.92; *p* = 0.0158) (Figure 4B). As CpG-ODN is a potent in vitro inducer of IL-10 production, B10 and B10_PRO_ cells were also assessed. Interestingly, despite the lower ratio of B10 cells in the peripheral blood of pregnant women, CpG-ODN activation was able to significantly augment the IL-10 production of B cells in pregnant women compared to the controls (2.15 ± 0.92 vs. 7.92 ± 1.35; *p* = 0.0002) (Figure 4B). As transitional B cells have been described to also secrete IL-10, their connection with IL-10^+^ B cells was analyzed, and we found that there is a positive correlation between these B cell subsets (*R* = 0.7818; *p* = 0.105) (Figure 4C). The expression of CD24 median fluorescence intensity (MFI) was significantly elevated in IL-10^+^ B cells compared to IL-10^−^ B cells in pregnant women (11.09 ± 2.06 vs. 17.14 ± 4.96; *p* = 0.0201). The activation of the PBMCs with CpG-ODN enhanced the CD38 expression (MFI) of IL-10^+^ B cells in comparison with IL-10^−^ B cells in pregnant women (1.93 ± 0.45 vs. 2.49 ± 0.58; *p* = 0.0089) (Figure 4D).

## 4. Discussion

Our findings indicated that the percentages of peripheral blood un-switched memory and transitional B cells significantly decreased in the third trimester of pregnancy. Moreover, the proportions of both CD5^+^CD1d^+^ Breg and IL-10-producing B10 cells also decreased, and these alterations may be crucial contributors to the physiological changes in the maternal immune status in the third trimester.

Prior studies have revealed that circulating B cells are reduced during pregnancy and the distribution of B cell subsets undergoes changes in healthy pregnant women. The decrease in peripheral blood B cells is most pronounced in the third trimester of pregnancy, due to the effects of the elevated estrogen levels on lymphopoiesis and the cellular migration into placental decidua [24]. Regarding the distribution of B cell subsets, a recent study reported increased percentages of naïve B cells and a decreased frequency of transitional B cells and un-switched memory B cells in the peripheral blood of women in late pregnancy, compared to non-pregnant women [20]. Our observations on transitional B cells and un-switched memory B cells are in line with these results. The importance of the redistribution of transitional B cells was underlined by a recent study, which shed light on the association between increased maternal blood transitional B cell frequencies and the development of allergy manifestation in progeny, suggesting the role of these cells in the Th1/Th2 bias observed in neonates [25]. The clinical significance of un-switched memory B cell abnormalities in pregnancy loss was also confirmed, although there are some controversial findings in the literature. Notably, a case report described a case of a woman suffering recurrent pregnancy loss and RA showing increased numbers of un-switched memory B cells [26], while another case report observed low levels of switched and un-switched memory B cells in a patient with a history of recurrent pregnancy loss and obstetric complications [27].

The redistribution of naïve and memory B cell subsets may be the consequence of decreased differentiation of naïve B cells into memory cells, since the high levels of progesterone present in late pregnancy inhibit B cell activation [28]. On the other hand, the reduction in transitional B cells is presumably caused by their migration to the maternal-derived decidua [20]. 

Besides the pregnancy-related modulation of the pro-inflammatory role of B cells, including antigen-presenting and antibody-producing activity, changes in the regulatory or suppression functions of maternal Breg populations are also crucial to avoid destructive immune responses. Among other functions, Bregs inhibit Th1 cell activation, suppress the differentiation and proliferation of Th17 cells, maintain Treg populations, decrease IFN-γ secretion, and reduce the accumulation of natural killer (NK) cells [29,30]. Breg cells, which represent no more than 10% of peripheral B cells, perform regulatory activity mainly through IL-10 production, although other mechanisms have also been described, including TGF-β production for promoting the generation of tolerogenic DCs and direct interaction with activated T cells via cell-to-cell contact [31,32]. Since no specific membrane markers or transcription factors have been identified yet, there is still no consensus on the exact markers of Breg detection. Based on the expression of various surface molecules, a number of Breg populations have been reported, including CD19^+^CD24^+^CD38^+^ B cells, CD19^+^CD1d^+^CD5^+^ B cells, and CD19^+^CD24^hi^CD27^hi^ B cells; however, IL-10-production is still regarded as one of the most important characteristics of functional Breg cells [32,33]. 

In our study, we observed significantly decreased percentages of both CD5^+^CD1d^+^ and CD19^+^CD24^hi^CD38^hi^ Breg populations in the peripheral blood of women in the third trimester of pregnancy, compared to non-pregnant controls. Similarly, the ratio of IL-10-producing B10 cells decreased in pregnant women as well. Based on previous studies, the proportions of Breg populations show significant changes in pregnant women, which may be elevated or even decreased, according to the different stages of pregnancy [34]. At the onset of pregnancy, the frequency of the CD19^+^CD24^hi^CD27^+^ Breg population was reported to increase in the peripheral blood of healthy women, but not in the case of spontaneous abortions. Since almost 95% of CD19^+^CD24^hi^CD27^+^ Breg cells express the receptor for human chorionic gonadotropin hormone (hCG), the elevation of hCG during pregnancy may be a potent factor regulating the number and function of Breg cells [35]. As for the CD5^+^CD1d^+^ Breg population, its initial increase is fundamental for avoiding immunological abortion by increasing Treg cell proportions and maintaining DCs in an immature state in pregnant mice models [36]. Human studies also confirmed the importance of the initial increase in Breg cells in healthy pregnancies, and pointed out the crucial role of the down-regulation of peripheral blood IL-10-producing B cells in the pathogenesis of repeated implantation failure and recurrent pregnancy loss [37,38].

**Figure 5:**
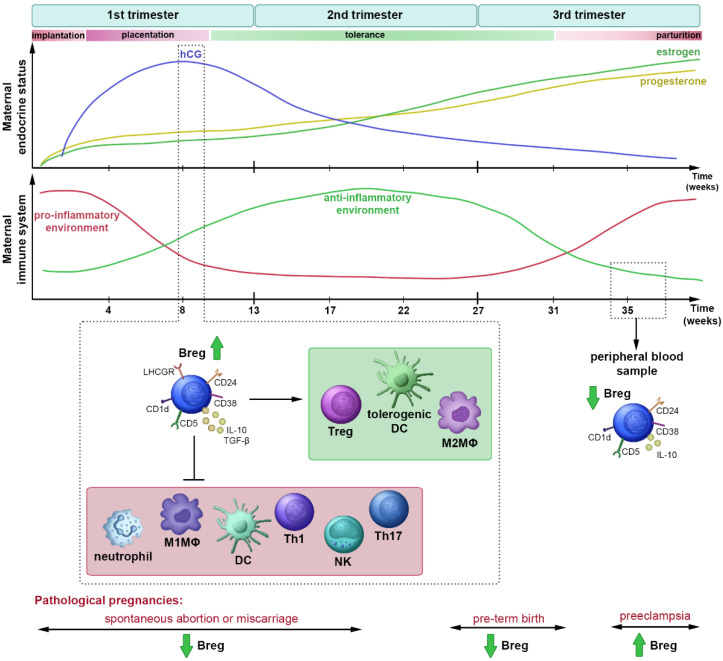
Changes in maternal endocrine and immune status during pregnancy based on current literature data and our results [41,42,43]. There are several cascading immune events, the nature and timing of which are crucial for a healthy pregnancy outcome. The first trimester of pregnancy is associated with inflammation, which is required for implantation and placentation. The second trimester is characterized by an anti-inflammatory immune status induced by hCG, which leads to Breg cell activation. Bregs suppress pro-inflammatory immune responses through the secretion of IL-10 and TGF-β, and induce tolerance via the activation of M2MΦ and Treg cells, as well as shifting DC differentiation in the direction of tolerogenic DCs, which establish fetal growth. In the third trimester, there is a switch to a pro-inflammatory immune state, which is necessary for parturition. Breg, regulatory B cell; DC, dendritic cell; Th, T helper; Treg, regulatory T cell; NK, natural killer; hCG, human chorionic gonadotropin; LHCGR, luteinizing hormone-chorionic gonadotropin receptor; M1MΦ, pro-inflammatory macrophage; M2MΦ, anti-inflammatory macrophage.

Nevertheless, in the third trimester of healthy pregnancy, we observed significantly decreased CD5^+^CD1d^+^ Breg proportions. Of note, a prior study shed light on the importance of CD5^+^CD1d^+^ Breg cells in the maintenance of pregnancy by reporting significantly increased peripheral blood CD5^+^CD1d^+^ Breg ratios during the third semester in preeclamptic patients, compared with those measured in women with normal pregnancy [23]. As for CD24^hi^CD38^hi^ Breg cells, a recent study also showed that their percentages of peripheral blood are significantly lower in late pregnancy, compared to the data measured in non-pregnant women [20], which is in line with our results on this cell subset. The Breg cell-mediated induction and maintenance of tolerance during pregnancy is summarized in Figure 5. Of note, despite the lower ratio of B10 cells in the peripheral blood of pregnant women, CpG-ODN activation was able to significantly augment the IL-10 production of B cells in pregnant women compared to those in the controls. However, the changes we observed in the expression of Breg subset-defining markers, namely, decreased CD24 expression and further enhanced CD38 expression, suggest that in vitro-induced IL-10-producing B cells may not be a dedicated subgroup of Breg cells [39]. The significant decrease in circulating Breg cells in the third trimester of pregnancy may be due to their increased migration into the growing mass uterus, which accumulates IL-10-producing B cells [40]; however, the full mechanism behind the activation, expansion, and reduction of Bregs during pregnancy still remains unclear.

Regarding the strengths and limitations of this study, in order to obtain a homogenous sample and reduce potential bias, we only enrolled subjects with singleton pregnancies between 34 and 37 weeks of gestation, and even with this small sample size, our results showed significant differences among B cell subpopulations. Nevertheless, more research is needed to clarify the role of Breg populations in detail as well as their interactions with the other elements of the immune system, in order to reveal the mechanisms of tolerance induction and the maintenance of pregnancy. The deeper understanding of B cell functions in pregnancy may have an important role in preventing maternal infections and optimizing maternal vaccination as wells.

## Data Availability

Datasets acquired for this study are included in the article. For further inquiries, contact the corresponding author.
